# Printed circuit board substrates derived from lignocellulose nanofibrils for sustainable electronics applications

**DOI:** 10.1038/s41598-025-91653-1

**Published:** 2025-03-08

**Authors:** Yuliia Dudnyk, Pavel Kulha, Václav Procházka, Gustav Nyström, Thomas Geiger

**Affiliations:** 1 Cellulose and Wood Materials Laboratory, Empa – Swiss Federal Laboratories for Material Science and Technology, Dübendorf, Switzerland; 2 PROFACTOR GmbH, Steyr, Austria

**Keywords:** Electronic devices, Electronic properties and materials

## Abstract

This study investigates lignocellulose nanofibrils (LCNF) as a sustainable alternative material for printed circuit board (PCB) substrates, demonstrating an application through the development of an eco-friendly computer mouse demonstrator. LCNF is derived from lignin-rich cellulose pulp, a side stream product of biorefinery processes, combining the natural strength of cellulose fibrils with lignin to enhance mechanical and electrochemical properties. The research outlines the process of fibrillating lignin-rich cellulose pulp at 10 kW/h per kg into LCNF, followed by thermal and pressure treatment (at Δp = 50 – 1500 kN, ΔT = 30 – 120 °C) to achieve a rigid PCB substrate. Comprehensive characterization of the LCNF substrate included assessments of its mechanical properties (flexural and tensile testing), dimensional stability, electrical properties, surface uniformity and thermal conductivity. The LCNF PCB was integrated in a computer mouse demonstrator featuring inkjet printing of circuit layouts and electronic component assembly, while the mouse housing was designed and 3D-printed using eco-friendly Wood-PLA filament. Electrical properties characterization of the printed circuit and resulting functionality of the computer mouse showcases a sustainable approach to eco-electronics using wood-derived materials. This study underscores the potential of wood-derived nanomaterials like LCNF to reduce electronic waste (e-waste) associated with conventional PCB materials and promote the development of a more eco-friendly electronics, contributing to sustainable, high-performance ecoPCBs and advancing green technology.

## Introduction

The growing issue of electronic waste (e-waste) presents a significant environmental challenge, primarily due to the widespread use of petroleum-based polymers and toxic heavy metals^1^. The rapid increase in e-waste production, driven by population growth and the digitalization of lifestyles, has been alarming. In 2022 alone, a record 62 million tonnes of e-waste were generated globally, marking an 82% increase from 2010, yet only 22.3% of it was properly recycled. Projections suggest that e-waste could increase by an additional 32% or more in the coming decades^2^. Recycling e-waste is a complex and costly process, due to the variety of materials, shapes, and active components involved.

Consequently, developing biodegradable and recyclable electronics, particularly those utilizing wood-derived polymers like cellulose, has garnered significant attention^3^.

Cellulose, the most abundant biodegradable and renewable polymer on Earth, offers a promising pathway to mitigate the environmental impact of e-waste. Its nanostructured forms, such as cellulose nanofibrils (CNFs), have emerged as an interesting material due to their exceptional mechanical properties through high surface area interaction, biodegradability, and sustainability making them suitable for various applications, including eco-electronics^3–7^. Besides, their thermal stability ensures performance integrity under varying temperature conditions, essential for electronic device substrates^1,6,8^.

In addition to its mechanical benefits, CNF has demonstrated potential also in electronic applications. Research on CNF in applications like 3D-printed supercapacitors^9^, energy storage devises^4^ and coloured cellulose displays^10^ highlights its versatility in both energy storage and display technologies, suggesting that CNF-based PCBs could support a wide range of CNF-supported electronic functions. In general, all PCBs can be separated in three different components, which are mechanical support substrate (passive component), active component (semiconductors, resistors etc.) and adhesives/inks^11^. In our study we are focusing on the mechanical support function which in conventional PCBs typically is provided by glass fibre-reinforced epoxy, materials that are challenging to recycle and require substantial energy for disposal^12^. Replacing these hazardous thermosetting polymers with biobased and biodegradable materials is crucial for mitigating E-waste and contributing to achieving net-zero pollution goals. Various materials have been investigated for their potential to replace conventional substrates, offering not only biodegradable properties but also favourable mechanical and electrochemical behavior^13^. Among these, silk protein^14,15^, cellulose acetate (CA)^3^, polylactic acid (PLA)^16,17^, and gelatin^18,19^ have shown promising results. While these materials provide eco-friendly alternatives, they still face performance limitations, particularly in areas requiring high mechanical strength or electrochemical stability under certain conditions^1,13^.

Based on prior research utilizing CNF for substrate development^20^, this study investigates the properties and potential of ligno-cellulose nanofibrils (LCNF) as a novel material for eco-friendly printed circuit boards (ecoPCBs). LCNF is produced from lignin-containing cellulose pulp, a side-stream product of the biorefinery process Fabiola™^21^ following fibrillation, the material produces a distinct CNF fraction that retains its natural bonding to lignin. This lignin-cellulose integration is expected to confer advantageous properties during substrate fabrication (hot-pressing) and has demonstrated strong mechanical and electrochemical characteristics through experimental analysis.

**Fig. 1:**
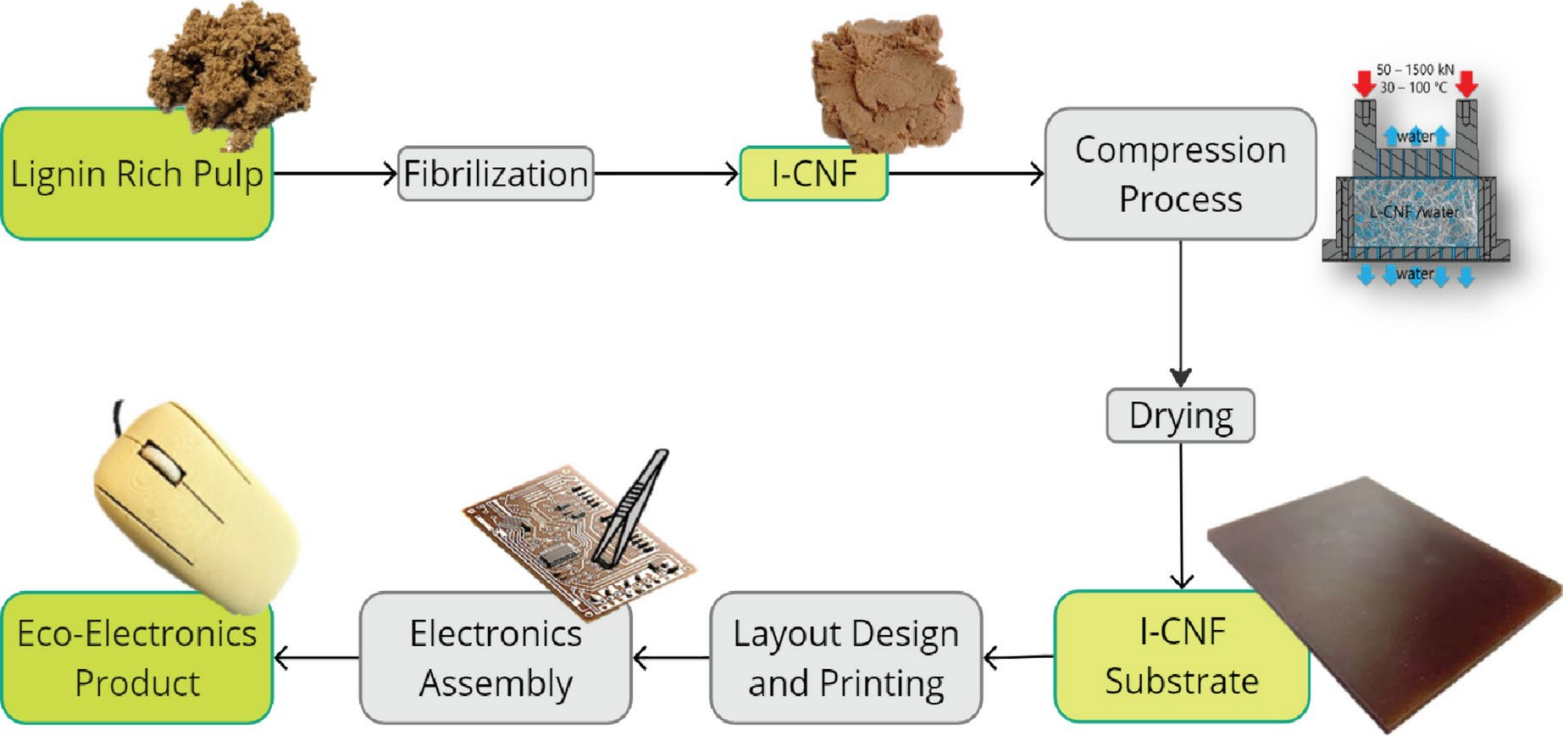
Schematic overview of the lignin-rich pulp fibrillation process into LCNF, followed by thermal and pressure-based processing to produce a rigid LCNF substrate. This substrate undergoes inkjet printing and electronic component assembly to create an eco-friendly computer mouse demonstrator, showcasing the results of this study.

To demonstrate the practical application of LCNF substrates in electronics, a computer mouse demonstrator was developed (Fig. 1). The mouse housing was designed and 3D-printed using biodegradable Wood-PLA filament, incorporating an LCNF-based printed circuit with assembled electronic components. This fully functional, eco-friendly computer mouse exemplifies the potential of wood-derived materials in advancing sustainable electronic devices.

## Materials and methods

### Materials

The fractionated pulp containing lignin 14% for LCNF preparation and high molecular weight beech wood lignin was kindly provided by TNO (Netherlands Organisation for Applied Scientific Research) from the established bio-refinery Fabiola™ process ^21^.

The CNF Valida S231C 8% for comparison methods was obtained from Sappi Maastricht BV (The Netherlands).

A stabilizing chamber or desiccator with 0% RH at 23 °C conditions was generated with using phosphorous pentoxide (P_2_O_5_) from VWR International GmbH.

### Preparation of LCNF substrate

#### LCNF fibrilization

This lignin containing pulp was disintegrated to LCNF using a Masuko MKZA10-20J CE friction grinding machine using a 10 kW/h per kg of pulp energy input which resulted in a finely fibrillated LCNF material.

#### LCNF substrate preparation

For preparation of 2 mm thick 15 cm diameter discs of LCNF substrate, approximately 44 g of LCNF dry mass obtained from 12.4% LCNF water suspension.

The weighed LCNF suspension was placed into a specially designed round stainless mould (D = 15 cm) (see Fig. 1 in the Supplementary information) for processing in a hot press (LAP150 Gottfried Joos Maschinenfabrik GmbH & Co. KG, Germany) using a modified process referring our previous work^20^. The design of mould was developed to increase the functional high pressure on the sample to get better material stiffness. The substrate forming process in the hot press was done in three steps – first, dewatering took place at 30 °C under vacuum (at Δp = 50 – 1500 kN for 40 min); second, drying at 80 °C under vacuum using porous metal mould plates (at Δp = 200 – 1500 kN for 40 min); third, hornification process^22^ and surface finishing at 120 °C with using polished metal plates (at Δp = 200 – 1500 kN for 15 min 30 s) was carried out.

Prepared substrates were post-dried in Vacuum Drying Oven VT6060 M-BL at < 2 mbar and 100 °C for 24 h under 10 kg weight between round metal porous disks to prevent any undesirable shape deformations.

#### Sample preparation

The dried disks were used to prepare samples for future testing using laser cutting (Thunder Laser Cutter Nova24—60W CO_2_) with 100% power and 27 mm per sec speed. Cut samples of LCNF substrate were stored in climate chambers at 50% RH (relative humidity) at 23 °C, 85% RH at 20 °C and 0% RH at 23 °C conditions for the future mechanical, analytical and electro chemical characterization.

### Preparation of computer mouse housing

The design of computer mouse housing was developed in our laboratory using CAD application software Rhino 7 (McNeel Europe SL), and optimized for printing on 3D printer model: Ender-5 S1 from Creality (China) with nozzle size of 0.6 mm, nozzle temperature 190 °C, bed temperature at 60 °C, layer height of 0.2 mm. G-code was performed with the slicing software PrusaSlicer version 2.8.0 (Prusa Research). Computer mouse housing model was printed using PLA-wood filament (Wood natural 1.75 mm from eSUN (China)) to increase the percentage of natural-based material in the device.

### Characterization and testing

#### Scanning electron microscope (SEM)

To analyze the fibrilization homogeneity of the LCNF and the surface structure of the densified substrates in detail, both top and cross-section views were examined using a Scanning Electron Microscope (FEI Quanta). The SEM was operated with an accelerating voltage of 5 kV and a working distance of 10 mm. Samples were mounted on SEM samples holders using double-sided carbon tape and coated with a 7 nm layer of platinum using a high vacuum sputter coater (Baltec Med020).

#### Fourier-transform infrared spectroscopy (FTIR)

ATR-FTIR spectra were collected using a FTIR Spectrophotometer IR Tracer-100 spectrometer equipped with a single-reflection attenuated total reflectance ATR with a diamond crystal QATR 10 Accessory Shimadzu (Switzerland). The samples were placed directly onto the ATR crystal, and the spectra were recorded in the range of 4000–500 cm⁻^1^ at a resolution of 4 cm⁻^1^. Each spectrum was an average of 64 scans to ensure a high signal-to-noise ratio.

#### Thermogravimetric Analyses (TGA)

Thermogravimetric analyses (TGA) of the LCNF were carried out using NETZSCH TG 209F1 Thermogravimetric analyzer from 30 to 800 °C at a heating rate of 20 K min^−1^ under a nitrogen atmosphere.

#### Flexural and tensile testing

The three-point bending test was performed to evaluate the flexural strength and the modulus of the LCNF substrate. The dimensions of the test samples were 10 ± 0.3 mm in width, 100 ± 0.3 mm in length, and 2 ± 0.3 mm in thickness. The test was conducted using a ZWICK Tensile Testing Machine ZwickRoell Z100 from ZwickRoell GmbH & Co.KG (Germany) with a span length of 40 mm and 1 kN load cell (F_min_ ≥ 1% F_nom_). The loading speed was set at 2 mm/min, and the flexural stress and strain were recorded until failure following standard DIN ISO 178.

The tensile properties of the LCNF substrate were assessed using samples also prepared via laser cutting. The tensile test samples had a dog-bone shape with a gauge length of 100 ± 0.3 mm, a width of 4.5 ± 0.1 mm, and a thickness of 2 ± 0.3 mm. The test was conducted on the same machine at a crosshead speed of 1 mm/min following DIN EN ISO 527–2 standard, using the clamping jaws up to max. 2.5 kN and load cell 100 kN. Key tensile properties, including tensile strength, Young’s modulus, and elongation at break, were determined from the stress–strain curves.

Five samples from each environmental condition were prepared for each measurement to obtain accurate mean and standard deviation.

#### Water vapor absorption

The water absorption and stability of LCNF samples under different environmental conditions were investigated using climate chambers. Two specific conditions were maintained: 50% RH at 23 °C and 85% RH at 20 °C. Before testing all samples were stored at 0% RH for 2 weeks to ensure no moisture presence. The samples were weighed at regular intervals using precise balances from Mettler Toledo MS with accuracy 0.1 mg to monitor changes in weight over time. Each environmental condition involved preparing three samples to ensure the accuracy of the results and to calculate the average and standard deviation.

#### Water absorption testing

The determination of water absorption values was performed following standard protocol IPC-TM-60 "Water Absorption, Metal Clad Plastic Laminates"^23^. Three samples (100 × 100 × 1.9) ± 0.3 mm^3^ were pre-dried in the convection oven at 105 °C for 5 h, then cooled to the room temperature at stabilizing chamber and weighted immediately upon removal from desiccator. Further, all samples were placed in a tank of deionized water and left for 24 ± 0.5 h at room temperature. At the end of time, samples were removed from water and all remaining water on the surface was wiped with paper towels and weighed immediately. The results were calculated with formula from protocol.

#### Dimensional Stability

Dimensional stability was detected following IPC-TM-650 standard IPC-TR-483 "Dimensional Stability Testing of Thin Laminates" protocol^24^. Three samples (100 × 100 × 1.9) ± 0.3 mm^3^ from 3 different environments (0%, 50% and 85% RH) had measuring points in the corners (F1, F2, W1 and W2). After measurement of the distances between these points samples were placed into convection oven at 105 °C for 4 h ± 10 min. Dried samples were placed into stabilization chamber for 1 h ± 5 min. Subsequently, the distances between the measuring points were again determined within 5 min. All samples were placed again into the oven at 105 °C for 2 h ± 5 min, then cooled in stabilization chamber for 1 h ± 5 min and measured distances between points.

#### Thermal Conductivity

Thermal conductivity measurements were performed on proprietary guarded hot plate device built on the base of EN 12,667 standard^25^ and described in the study for (60 × 60 × 12.5) mm^3^ samples. The device consists of a cooling plate at the bottom, followed by the sample and finally the guarded hot plate on top. During measurement, the tested sample was surrounded by a polyethylene insulation material to keep heat flow between the plates as vertical as possible^8^. The LCNF board was prepared in dimensions of (60 × 60 × 11.99) ± 0.3 mm^3^.

#### Electrical properties

The electrical resistance properties of the LCNF substrate were evaluated using a Keithley DMM6500 6.5 Digit Multimeter configured for four-point probe measurements. This method employed the four-wire technique to eliminate the influence of lead and contact resistance. Four equally spaced collinear probes were placed on the surface of the LCNF substrate: the two outer probes sourced current (I), while the two inner probes measured the voltage drop (V). Five samples with a width of 11.5 ± 0.8 mm, a thickness of 1.88 ± 0.3 mm, and a length of 100 ± 0.7 mm, from each environmental condition were prepared for measurement to calculate the average and standard deviation. The inner probe length is 1.9 mm.

The resistivity (ρ) of the samples was calculated using the formula^26^:

$$\rho =\frac{R (w\times t)}{L}$$

where R – resistance (value from multimeter measurements)$, \Omega \cdot cm$; L – length between inner probes, cm; w- width of sample, cm; t- thickness of sample, cm.

#### Surface uniformity analysis

The surface has been analyzed using Keyence VK-X3000 3D laser scanning microscope. LCNF substrate has been overprinted with silver nanocrystalline ink using Nanodimension´s Dragonfly IV®, using AME (Additively Manufactured Electronics) process.

## Results and discussion

**Fig. 2:**
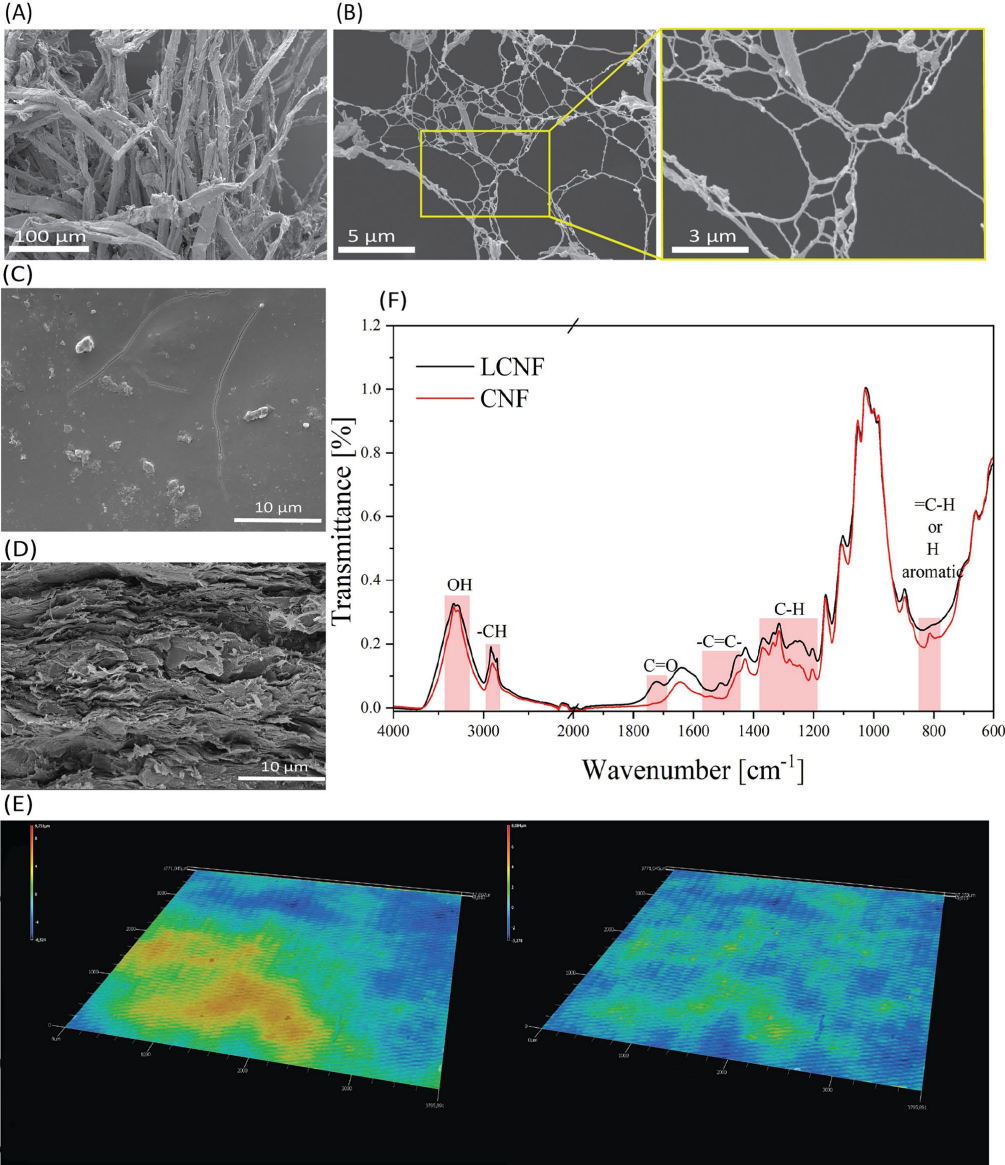
SEM images of lignin containing pulp (**A**) and LCNF (**B** with zoom), LCNF substrate from top (**C**) and cross-section at tensile break (**D**). On the images (**E**) shown Surface uniformity analysis of the printed circuit on LCNF substrate with laser profilometer. **F**) ATR-FTIR comparison analysis of LCNF and CNF.

Ligno-cellulose fibers are disintegrated into ligno-cellulose fibrils by high shear forces mechanical grinding in the presence of water. The degree and homogeneity of fibrillation is verified by electron microscopy. Figures 2A and 2B show images of fibers before disintegration and images of the fibrillated LCNF after processing. The cellulose fibers, originally several millimeters long and more than 10 µm thick (Fig. 2A), are disintegrated into a network of fibrils with lengths of several micrometers and diameters of a few nanometers (Fig. 2B). The absence of fiber residues and larger fibril agglomerates are an indication of complete disintegration of the fibers and, therefore, high homogeneity of the suspension.

As has already been shown in earlier studies on lignin-free CNF, the water that stabilizes the fibril network can be removed by pressure and temperature. This results in hornification, i.e., a reconstruction of the hydrogen and van der Waals bonding network of the cellulose with each other, and the fibrils densify to almost the theoretical density of 1.5 kg/cm^3^ of cellulose. Reaching this density in the case of LCNF requires the free accessibility of the surface of the fibrils their bonding interfaces as well as their mobility and reconfigurability in a densely packed network, both of which are given by the complete disintegration of the original fibers (Fig. 2B) but is partially hindered by the presence of lignin still in the system. After complete dewatering, densification and drying of the LCNF, a uniform stiff solid mass in the form of a round plate was formed (see Fig. 1). Electron microscopy of the surface and fracture surface of the plate provide information about its structure and internal morphology.

The surface of the densified LCNF substrate, which is later used as a substrate in the PCB application, is essentially non-porous and flat but covered with individual micrometer-sized particles (Fig. 2C). This surface structure is also significantly defined by the polishing quality of the steel mold. During dewatering of the LCNF suspension in the steel mold, the fibrils are oriented into layers by the outflowing water. In addition, surface uniformity of LCNF substrate was analyzed (Fig. 2E) to understand printability and assembly properties. Analysis shows that LCNF substrates are macroscopically flat which works very well with inkjet printing process, however, to achieve successful assembly of bare die chip the surface inequities needed to be tacked to obtain flat surface within area marked in yellow (Fig. 2E left-side image). The contact electrodes are equipped with higher bumps to assure contact with the electrode of bare die chip. Otherwise, in general electronics applications the surface uniformity of LCNF applicable.

The cross-sectional SEM images Fig. 2D provided insights into the internal structure of the LCNF substrates. The images show a layered cellulose fibrils deposition pattern, which is associated to the arrangement inducted by flow and hornification processes that occur during the substrate forming process. These processes involve the reorganization and bonding of cellulose fibrils, resulting in a dense, well-ordered stiff internal structure. The hornification process ^27^, which involves drying and re-bonding of the cellulose fibers, contributes to the mechanical robustness and dimensional stability of the substrates. This structured internal morphology not only enhances the mechanical strength of the substrates which exceeds required standards^28^ but also plays a vital role in their overall performance in the future electronic applications.

Lignin and hemicelluloses naturally presented in the starting fibers and resulting fibrillated materials have an influence on the physical and chemical properties of the LCNF substrate as well as its dewatering and hornification. An unquantified observation is that the dewatering rate of the LCNF suspension in the mold is higher than that of a lignin-free CNF suspension. It is assumed that the retention capacity of the cellulose is reduced by the attached more hydrophobic lignin, allowing the water to drain faster from the fibril network.

To understand the nature of the easier dewatering during the board forming process for LCNF compared to when lignin free CNF ^20^ is used ATR-FTIR measurements were performed to study the intramolecular bonding.

The ATR-FTIR spectra presented in Fig. 2F contain two samples: lignin free CNF and LCNF.

In the 3400–3200 cm⁻^1^ region, a broad peak is observed in both CNF and LCNF samples, indicative of O–H stretching vibrations commonly associated with hydroxyl groups (–OH) in cellulose^29,30^. The 3000–2800 cm⁻^1^ region shows weaker peaks attributed to C-H stretching vibrations, typical of aliphatic compounds present in cellulose and lignin^31,32^.

The peaks in the 1750–1690 cm⁻^1^ region are associated to C = O stretching and 1450–1580 cm^-1^ due to -C = C- stretching are related to aromatic skeletal vibrations typical for lignin and hemicellulose and weaker in lignin free CNF sample^29,33^. The 1200–1400 cm⁻^1^ region displays strong peaks in the CNF and LCNF samples, indicative of C-H stretching vibrations or angular strain of CH (cellulose and lignin)^34^. The peak below 900 cm⁻^1^ mainly in LCNF can be attributed to out-of-plane bending modes, often associated with aromatic rings in lignin^34^.

The ATR-FTIR analysis reveals that the presence of lignin in LCNF introduces aromatic structures, as evidenced by peaks in the 1750–1690 cm⁻^1^ and 1450–1580 cm⁻^1^ regions, corresponding to C = O and C = C stretching vibrations, respectively. These aromatic structures are associated with increased dewatering efficiency during the board-forming process compared to lignin-free CNF. Lignin’s polyphenolic and hydrophobic nature reduces the water-binding capacity of the fibrils by obstructing hydrogen bonding sites. Consequently, water retention within the fibril network is minimized, facilitating more efficient water removal and quickening the substrate formation process.

In electronic applications such as PCBs, the presence of moisture can significantly affect the performance and durability of the materials used. Moisture absorption can lead to reduced insulation resistance, increased dielectric constant, and mechanical degradation, which in turn impact the reliability of electronic components. When cellulose-containing materials, like LCNF, are used in eco-friendly PCB substrates, their inherent hygroscopic nature makes it essential to thoroughly examine its behavior for optimizing material properties to ensure stable performance in electronic applications.

**Fig. 3:**
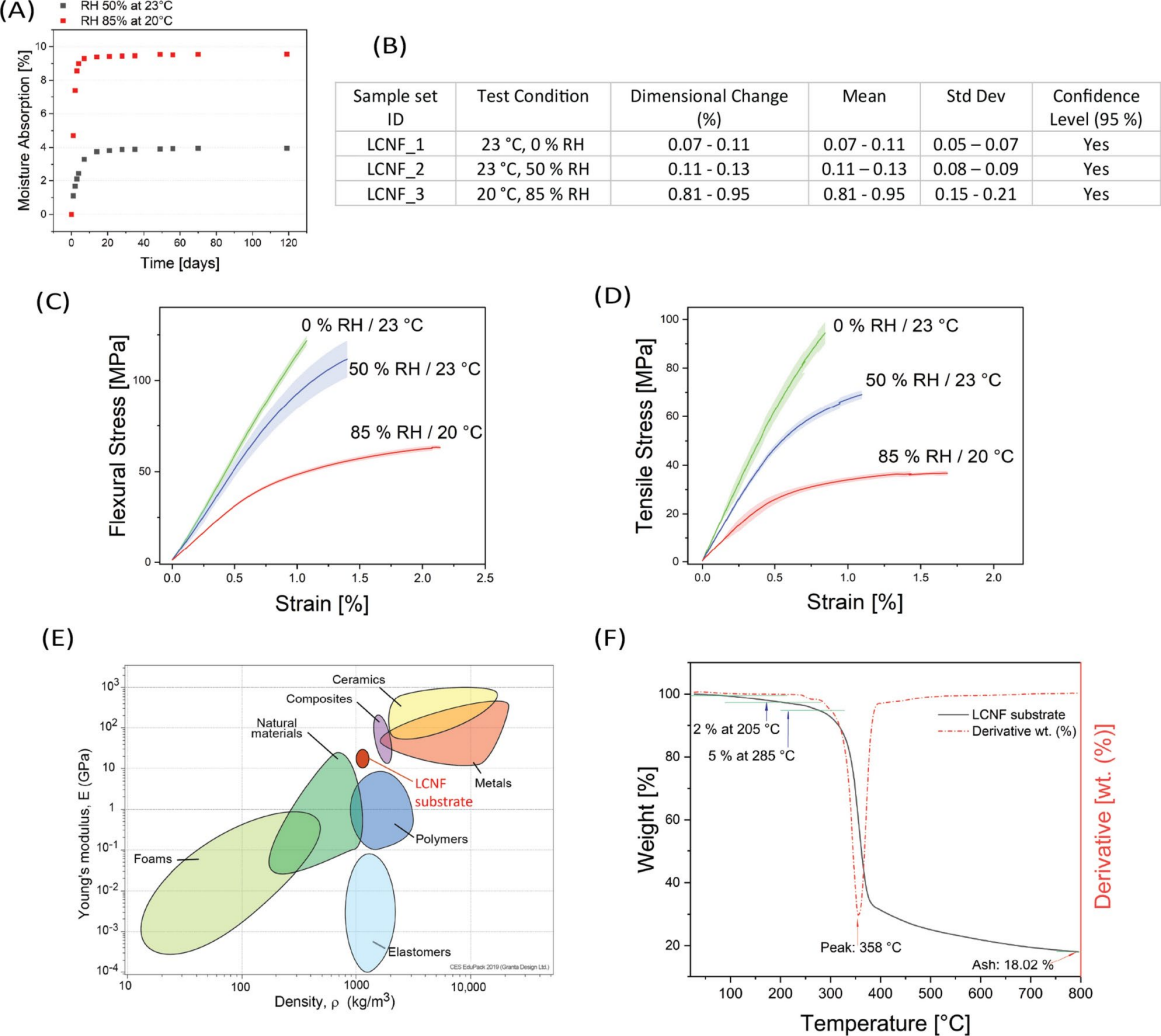
**A**) Moisture absorption of LCNF substrates at 50% RH 23 °C and 85% RH 20 °C environment conditions during time. **B**) Table shows results of the dimensional stability tests of LCNF substrates. **C**) 3-point bending test results of LCNFs substrates stored at various environmental conditions 0% RH and 50% RH at 23 °C, and 85% RH at 20 °C for 30 days to reach constant water vapor absorption values. **D**) Tensile test results of LCNFs substrate stored at various environmental conditions 0% RH and 50% RH at 23 °C, and 85% RH at 20 °C for 30 days to reach constant water vapor absorption values. **E**) Ashby plot of materials properties integrated with CNF and LCNF data results^7,23^. **F**) Thermogravimetric Analysis (TGA) results of LCNFs substrate.

To test the LCNF substrate stability at different environmental water vapour absorption tests were performed (see Fig. 3A).

The results indicated that LCNF substrate samples stored at 85% RH reached a constant water vapor absorption value more quickly than those stored at 50% RH. At equilibrium, samples stored at 85% RH absorbed approximately 9.5% moisture, while those stored at 50% RH absorbed up to 4% of water vapor. This faster stabilization at higher humidity levels suggests a more rapid equilibration process under conditions of higher moisture availability.

For a better understanding and to compare the properties of the LCNF substrate and conventional glass fiber reinforced epoxy substrates PCB substrates, liquid water absorption following the IPC-TM-650^23^ standardized test for electronics was carried out. Using the protocol, substrates with LCNF showed a 34.2% of moisture absorption (SD = 0.321), surpassing the minimum stated requirements of 5.6%^28^.

A result of the water uptake through vapor or liquid water immersion is that water changes the internal structure of the substrates through plasticization. In general, a loss of dimension stability must therefore also be expected in such systems. The evaluation of the dimensional stability for LCNF substrate was done in comparison to commercial PCB materials following the ICP-TR-483 standard^24^. Table on Fig. 3B shows the dimensional stability measurements for LCNF substrate samples. Each sample was subjected to a controlled environment, and the percentage change in dimensions was recorded. The mean values, standard deviations, and confidence levels at 95% were calculated. According to the standards the high-performance PCBs require dimensional stability under 0.1%, while for general electronic applications requirements in the range up to 0.5%^28^.

The significant difference in water absorption between the two environmental conditions has important implications for the use of LCNFs substrates in indoor applications. The higher water absorption at 85% RH indicates that LCNF substrates are sensitive to ambient humidity levels, which could affect their dimensional stability, mechanical properties over time and reduce insulation properties.

The LCNF_1 sample set exhibited a dimensional change from 0.07% to 0.11% under the test conditions of 23 °C and 0% RH, with a mean of 0.07—0.11% and a standard deviation of 0.05—0.07%. This sample set fall within the 95% confidence level, indicating high statistical reliability. The same behavior showed samples set LCNF_2 stored at 50% RH and 23 °C with dimensional change of 0.11 – 0.13%.

However, LCNF_3 sample set stored at 20 °C and 85% RH shows a dimensional change of 0.81% to 0.95%, this sample significantly exceeds the typical standard values^28^, indicating substantial instability in high humidity conditions.

In bio-based materials, such as cellulose, polyesters, polyamides, etc., the plasticizing property of water molecules, as soon as they penetrate the inner structure of the material, also becomes noticeable through a reduction in its mechanical strength and modulus. To investigate this effect, the mechanical properties under bending and tension of various LCNF substrates were analyzed after storage at different humidity levels and temperatures.

The results of the three-point bending test (Fig. 3C) showed that LCNF substrates have significant flexural strength and modulus, indicating good resistance to bending forces. The average flexural strength was found to be 133.25 ± 9.9 MPa, and the flexural modulus was 11.33 ± 0.6 GPa for samples stored at 0% RH 23 °C. Conditioning at 50% RH 23 °C resulted in the reduction of the flexural strength to 114.27 ± 11.2 MPa and the flexural modulus to 9.94 ± 2 GPa. A significant reduction of the mechanical properties was observed for the samples stored at 85% RH and 20 °C. The flexural strength value dropped to 63.23 ± 1.7 MPa and the flexural modulus to 6.22 ± 0.2 G Pa. These values suggest that the LCNF substrate can effectively withstand mechanical stresses encountered in typical electronic applications and exceeds significantly minimum requirements which is 83 N/mm^2^ (83 MPa) for cellulose reinforced PCBs and 415 N/mm^2^ (415 MPa) for epoxy glass fiber reinforced PCBs ^28^.

The LCNF substrates are a multi-material compound of fibrils, lignin, hemicelluloses, and water. In previous studies on lignin-free CNF substrates, a flexural strength of 200 MPa and a flexural modulus of 11 GPa were measured^20^, whereby the sample was previously conditioned at 50% RH and 23 °C. The LCNF substrates achieve lower values for strength and modulus under the same conditions, which is likely due to the lignin and hemicelluloses^21^.

The same trend in the measured values can also be observed in tensile tests on LCNF samples at different humidities and temperatures (see Fig. 3D). Samples of the LCNF substrate showed a tensile strength of 95.3 ± 4 MPa, a Young’s modulus of 13.05 ± 0.8 GPa, and an elongation at break of 1.2% for the samples stored at 0% RH 23 °C. Samples that was stored at 50% RH 23 °C showed in average a tensile strength of 68.64 ± 3.2 MPa, a Young’s modulus of 10.2 ± 0.5 GPa, and elongation at break of 1.36%. Mechanical properties of the samples that were stored at 85% RH 20 °C however shows drastic reduction of the tensile strength to 36.94 ± 0.6 MPa, a reduction of Young’s modulus to 5.98 ± 0.7 GPa, with an elongation at break of 1.87%.

The measured strengths and moduli are compared with the properties of technical plastics and fiber reinforced composites. Ashby diagrams are a useful tool to compare material properties and identify material classes rapidly and effectively. Fig. 3E shows an Ashby diagram with modulus and material density values of different material classes including the measured values of LCNF substrates assuming the density is 1484 kg/m^3^. At this density, LCNF substrates achieve a comparable stiffness to various lighter woods and the heavier composite materials used for PCBs. In contrast, the stiffness at the same density of LCNF is significantly higher than that of technical polymers.

TGA is important for PCB material substrate characterization to evaluate the thermal stability and decomposition behavior, determining the material’s resistance to the elevated temperatures during processing and operation. In Fig. 3F the TGA analysis results shows that the LCNF substrate undergoes an initial weight loss due to moisture evaporation till 100 °C followed by a substantial degradation phase starting around 205 °C initiating 2% weight loss and 5% weight loss appears at 285 °C followed with a peak of full decomposition at 358 °C. The final residue, about 18.02% of the initial weight suggests the presence of non-volatile inorganic materials and not completely pyrolyzed carbon due to the nitrogen atmosphere.

The thermal conductivity of the LCNF substrate was measured on proprietary guarded hot plate devise showed results in the range of 0.245—0.302 ± 0.4 W/mK. This falls within the Level A^28^ standard requirements for PCB materials (see Supplementary Information Table 1). For comparison, previous studies have shown that epoxy glass fiber-reinforced substrates typically exhibit a thermal conductivity of approximately 0.343 W/mK under standard conditions^35^.

The resistance ($\rho$) for LCNFs stored in different environmental conditions for 30 days was determined as follows:

$${\rho }_{\text{LCNF at }0 { \% RH}}$$= 23.9 × 10^3^ $$\Omega$$ ·cm.

$${\rho }_{\text{LCNF at }50{ \% RH}}$$= 14 × 10^3^ $$\Omega$$ ·cm.

$${\rho }_{\text{LCNF at }85 { \% RH}}$$ = 9 × 10^3^ $$\Omega$$ ·cm.

These samples exhibits excellent performance according to the standard for cellulose reinforced systems which is 10^3^ $\Omega$ ·cm^28^. For comparison, FR4 epoxy glass fiber reinforced PCB substrates typically exhibit a volume resistance of approximately 10^8^ – 10^9^ Ω·cm under similar conditions^28^.

The measurements showed consistent resistance values across different samples, indicating homogeneous material properties and effective processing techniques. Nevertheless, LCNF substrates exhibit lower but competitive resistance compared to FR4 substrates at all tested humidity levels. The resistance of LCNF substrates decreases significantly with increasing humidity. This indicates that LCNF substrates based on CNF can potentially match the electrical performance of FR4 substrates under specific environmental conditions, supporting their viability as sustainable alternatives in the electronics industry.

## Demonstrator

Our results indicate a high potential of using these biodegradable LCNF substrates as PCB substrates in electronics applications, which could potentially comply with existing regulations for indoor electronics. Inspired by these findings, we embarked on the ambitious project of integrating our green PCBs in consumer electronics application in the form of the biodegradable computer mouse.

The concept of a fully biodegradable mouse is ambitious, but a significant step forward can be achieved with a system designed entirely from eco-friendly, biodegradable PCB substrate and mouse cover materials. Regarding the electronics components, after complete disintegration of the substrates, we foresee a recycling pathway where it will be possible to collect and recycle these parts for future use^20^.

The PCB layout design was adapted from an assembly kit borrowed from NAGER IT e.V. (Germany)^36^, along with the electronic parts by PROFACTOR GmbH (Austria), and employed the LCNF substrate to print and assemble the electronic parts, making the demonstrator operational.

### Inkjet printed interconnections

**Fig. 4:**
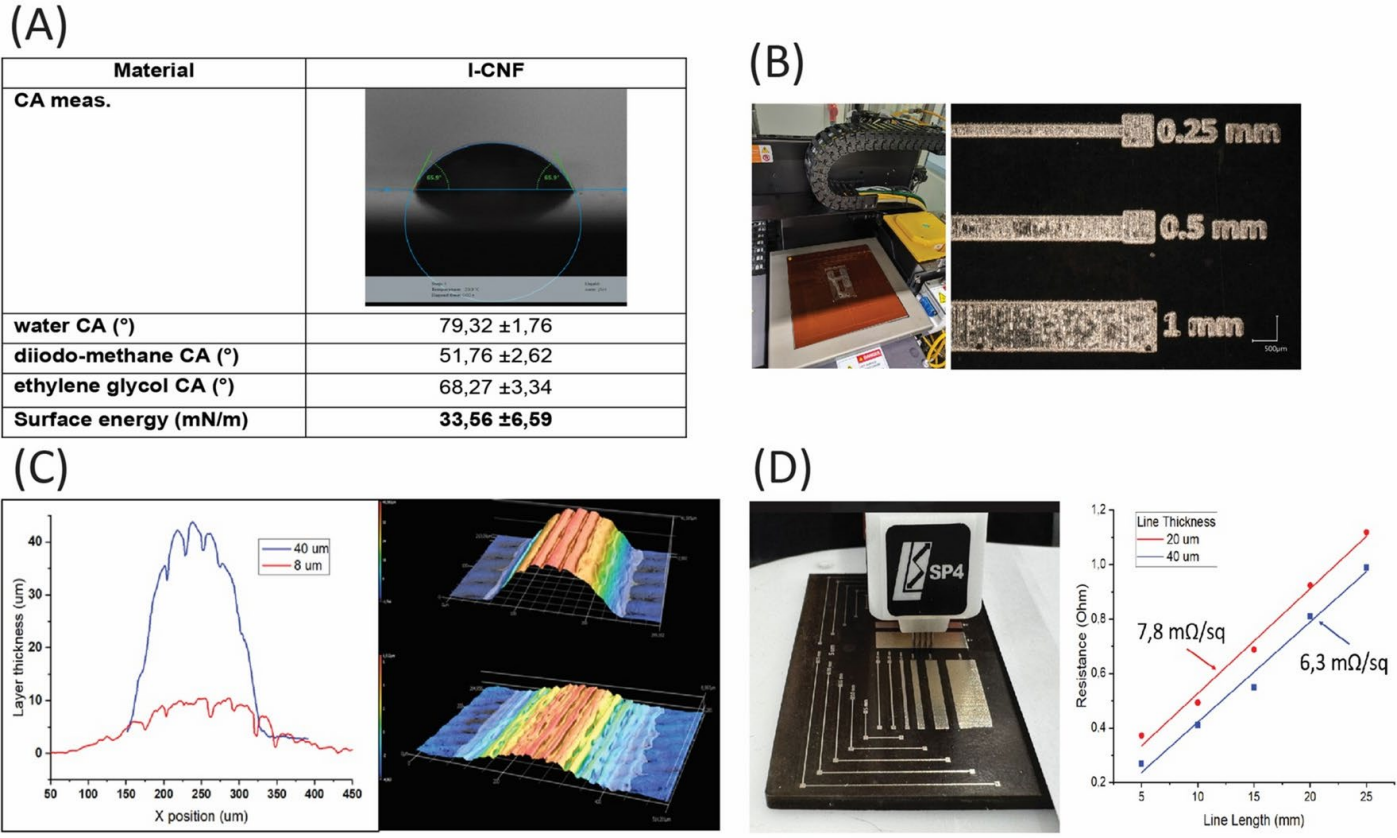
**A**) Contact Angle Measurements (CA meas.) and Energy calculations table. **B**) Test LCNF board loaded in the DragonFly printer (left), test structure printed on the board to estimate quality of printing and resistance of printed interconnections. **C**) Profiles of conductive layers printed on LCNF substrate, the 3D profile view was taken in the printing direction. **D**) Test station for resistance measurements (left), line resistance vs line length (right).

To understand the behavior of the ink on the substrate, surface contact angle measurements and calculation of the surface energy were performed on the LCNF substrate (Fig. 4A). A good match between surface energies of the surface and ink was observed (33.56 ± 6.59 mN/m), which was subsequently confirmed by printing results that exhibit good quality (sufficient resolution, no significant de-wetting or spreading of the ink on the LCNF substrate).

LCNF substrates were wiped with ethanol to remove grease and dust particles before loading to the printer. The printing pattern was adapted from an open-source project, “Fair Mouse,” from NAGER IT^36^. Minor modifications of the pattern have been done to meet printing process requirements. Before printing the layout for the sustainable mouse test structures were printed on the LCNF substrates to estimate the adhesion and resistance of printed layers. The printing process includes inkjet deposition of conductive ink layer by layer followed by inline NIR drying of the ink to evaporate the carrying solvent and sintering to achieve good conductivity (Fig. 4B). The LCNF substrate was kept at 120 °C (tray temperature) during printing.

### Characterization of printed lines

To confirm that a good resistance of the printed track can be achieved the conductive traces on LCNF substrate were characterized. Layers with thicknesses up to 40 µm were fabricated and their 3D profiles were analyzed (see Fig. 4C). Representative profiles of layers with thicknesses of 7 µm and 40 µm were characterized using a Keyence VK-X3000 laser scanning microscope. Electrical resistance of the printed tracks was evaluated using a custom-designed four-probe measurement system, comprising a Keithley 2400 source meter and a Keysight 34465A multimeter configured for 4-wire measurements. Interconnections with a thickness of 40 µm exhibited a sheet resistance as low as 6.3 mΩ/sq. For the demonstrator board, interconnections with a thickness of 20 µm were selected to balance printing time and sufficient electrical conductivity. Line resistance measurements for various line lengths, performed on tracks with a width of 150 µm, are presented in Fig. 4D.

### Assembly of electronic components

**Fig. 5:**
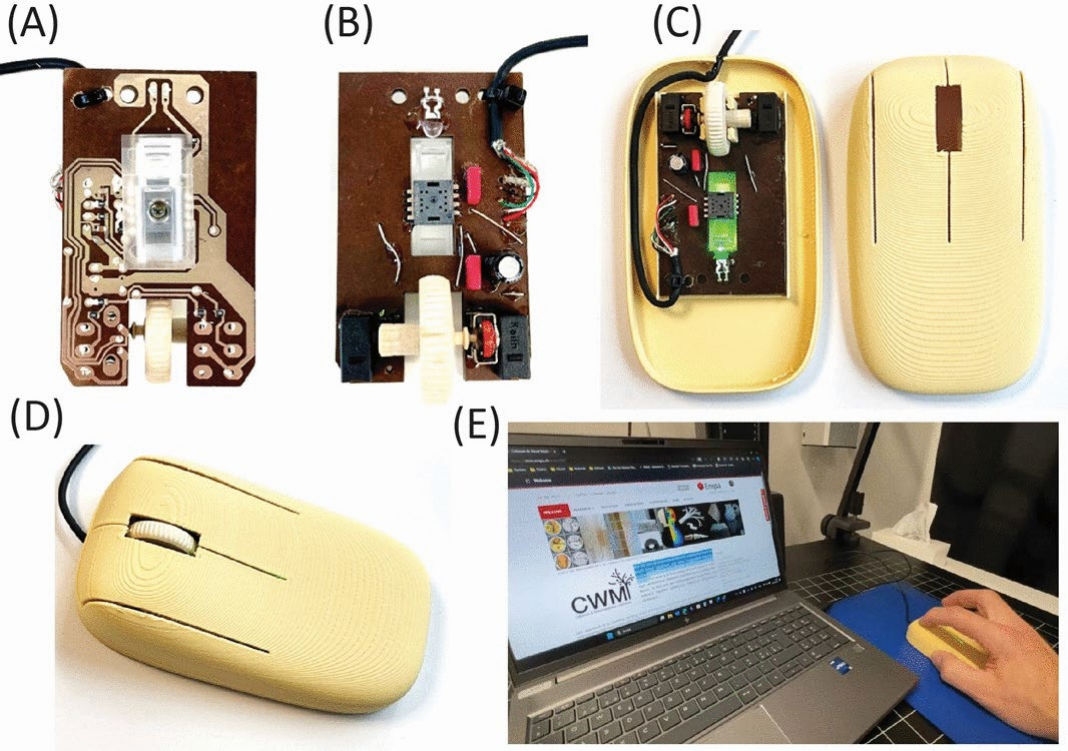
LCNF ecoPCB with assembled components, (**A**) bottom and (**B**) top view. On (**C**) shown LCNF ecoPCB nested into 3D printed PLA-Wood housing and (**D**) fully assembled functional eco-mouse demonstrator. **E**) working sustainable computer mouse demonstrator at work.

The assembly of passive components on the inkjet-printed circuit, built on a lignin-based PCB, was carried out manually. To ensure the mechanical stability of the electronic components, a commercially available UV-curable adhesive was applied to fix the electronic components on the top of the substrate. Electrical connections were made by glueing the components on the bottom (interconnections) side using a low-temperature cured conductive paste (Henkel PF050) (Fig. 5A). Curing to establish good electrical contacts was done in convection oven at 120 °C for 30 min. The functionality test of the whole assembly was done simply by attaching a USB cable and connection to a USB port in a PC.

The fabricated sustainable printed circuit board (Fig. 5A,B) was tested and found fully functional after placing it in a 3D printed housing out of PLA-Wood filament (Fig. 5C-E), as it is demonstrated in the video in the supplement information.

The development of this first working demonstrator, which is 70 wt% based on eco-friendly material of which 28% is wood materials, represents a significant advancement in the creation of eco-friendly devices. This demonstrator not only showcases the feasibility of using biodegradable substrates in electronics but also sets a precedent for future innovations in sustainable technology.

## Conclusions

This study introduces an innovative approach to fully biobased PCBs by using hot pressed wood-derived LCNFs as substrate material. The presence of the lignin in LCNF enhances the dewatering process during the PCB manufacturing compared to lignin-free CNF, potentially increasing its industrial appeal.

The LCNF substrates exhibit exceptional mechanical properties, with a Young`s modulus around 11 GPa, overperforming a number of natural materials and polymers. Additionally, they demonstrate good electrical performance under controlled environmental conditions and strong compatibility with conventional printing techniques, making them highly suitable as PCBs for eco-friendly electronics.

The integration of LCNF into a functional computer mouse demonstrator, prepared by 3D-printing PLA-wood filaments, serves as a proof of concept and highlights the potential of LCNF as a sustainable alternative to traditional PCB materials. However, high environmental humidity reduces the performance of LCNF substrates, increasing conductivity of the substrate, and reducing dimensional stability and mechanical properties. These factors can negatively impact the performance of LCNF PCBs, reducing device robustness outside of controlled environment (over 50% RH) and indicates a need for additional green humidity protection and stabilization treatments.

Future research should focus on enhancing the dimensional stability and electrical performance of LCNF substrates under extreme environmental conditions and high humidity. Developing scalable and cost-effective production methods will further boost their competitiveness as a PCB replacement.

Potential applications include consumer electronics, medical devices, and other indoor systems prioritizing reduced environmental impact. By introducing a biodegradable PCB platform, this work contributes to reducing electronics’ ecological footprint and advancing a circular, sustainable industry.

## Supplementary Information

Supplementary Information 1.

Supplementary Video 1.

## Data Availability

The datasets used and/or analyzed during the current study available from the corresponding author on reasonable request.
