# Supplementary material for: Printed circuit board substrates derived from lignocellulose nanofibrils for sustainable electronics applications
Source: Sci Rep. 2025 Mar 8;15:8080. doi: 10.1038/s41598-025-91653-1 (PMC11890781; doi:10.1038/s41598-025-91653-1)
Supplement: Supplementary file 1 — Supplementary Information 1. [file 41598_2025_91653_MOESM1_ESM.docx]

**Supplementary Information for**

**"Printed circuit board substrates derived from ligno-cellulose nanofibrils for sustainable electronics applications"**

Yuliia Dudnyk^1^, Pavel Kulha^2^, Václav Procházka^2^, Gustav Nyström^1^* and Thomas Geiger^1^*

^1^ Cellulose and Wood Materials Laboratory, Empa – Swiss Federal Laboratories for Material Science and Technology, Dübendorf, Switzerland

^2^PROFACTOR GmbH, Steyr, Austria

*Corresponding authors: Thomas.Geiger@empa.ch, Gustav.Nystroem@empa.ch


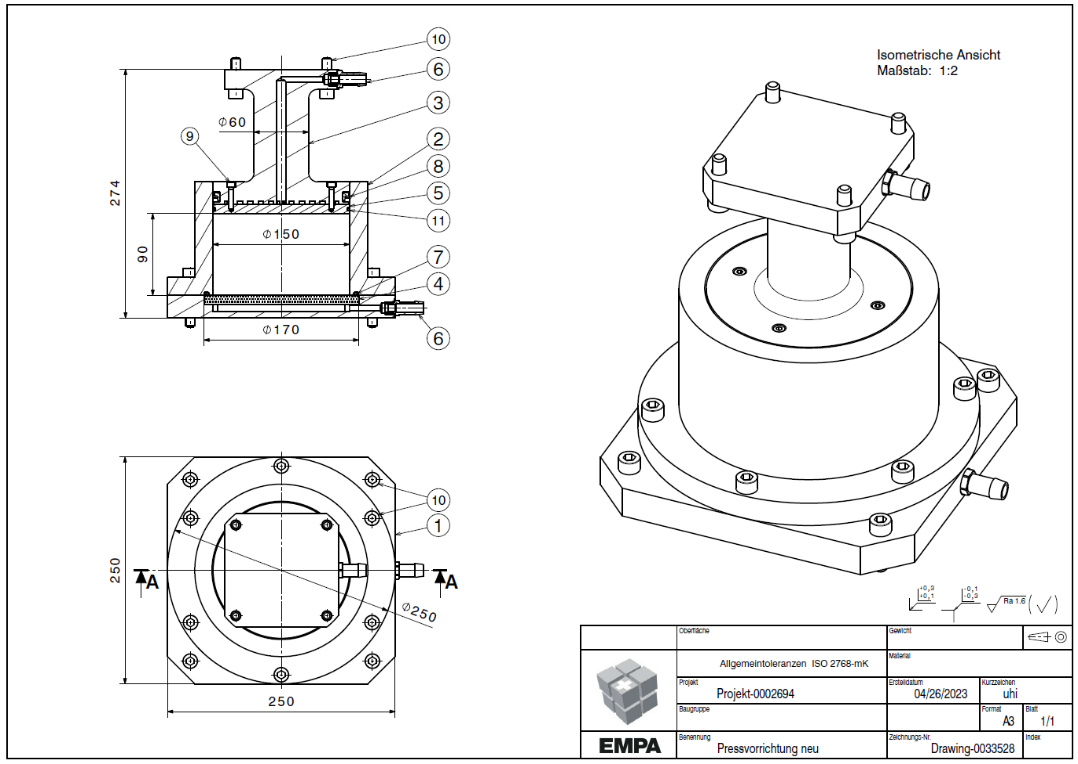


*Figure 1. Technical drawing of round mold for the LCNF board forming.*


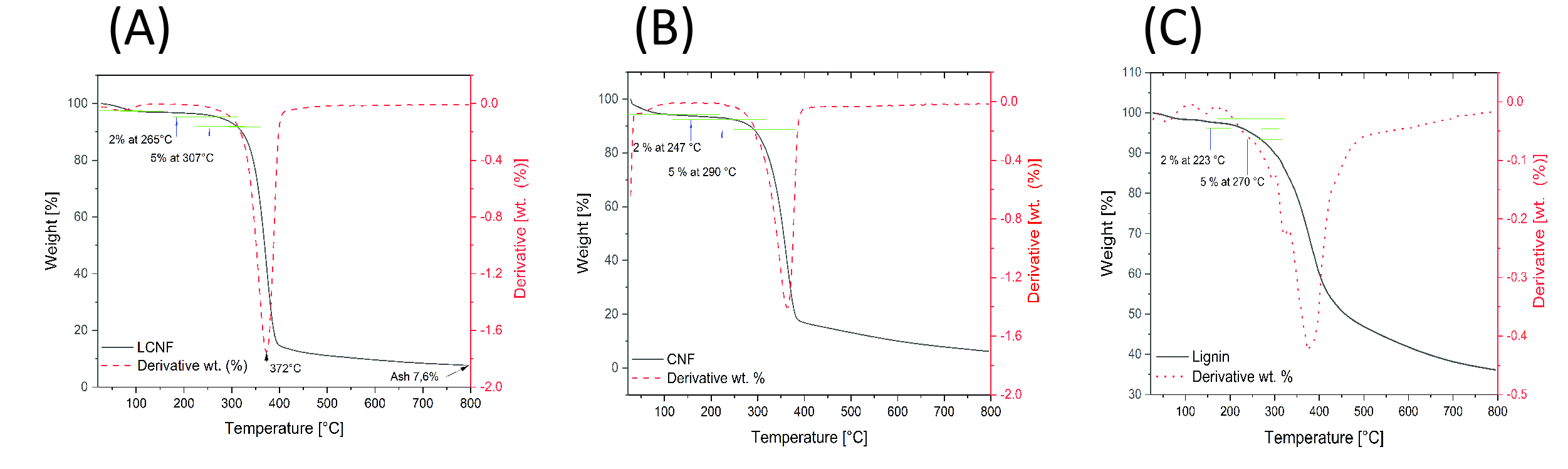


*Figure 2. TGA (Thermo-Gravimetric Analysis) comparison of disintegration of LCNF (A), CNF (B) and lignin (C).*

*
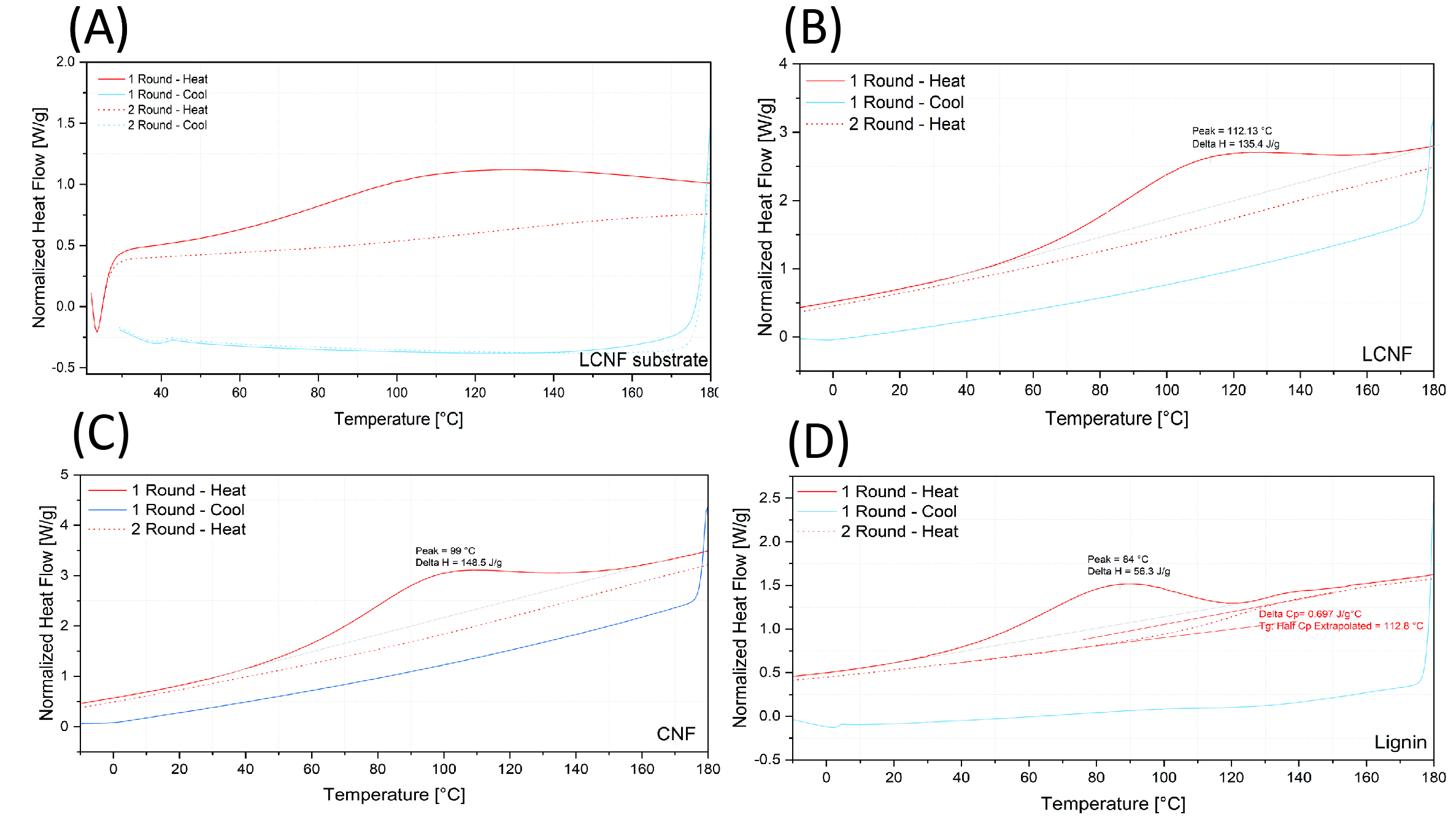
Figure 3. DSC comparison analysis of (a) LCNF substrate, (b) LCNF, (c) CNF and (d) Lignin*

Table 1. Comparison summary of standard requirements.

| Standard Requirement | LCNF substrate | Cellulose paper reinforced substrate | Epoxy glass fiber reinforced substrate (FR4) |
| --- | --- | --- | --- |
| Water absorption (max. 5.6 %) | 34.2 % | 1 % | 0.5 % |
| Flexural strength | 133 MPa | 83 MPa | 415 MPa |
| Thermal conductivity (according to manufacture requirements, however should meet one of following levels:  Level A ≤ 1.0  Level B > 1.0 ≤ 2.0  Level C > 2.0 ≤ 3.0  Level D > 3.0 ≤ 5.0) | Level A | Level A | Level A |
| Volume resistivity | 14 × 10³ $Ω$ ·cm | 10³ $Ω$ ·cm | 10^8^ – 10^9^ Ω·cm |
| Dimensional Stability –   - 1. % - for high performance electronics,   0.5 % - for general electronics application  0.5 – 1 % - non-applicable | 0.1 % | 0.1 % | 0.1 % |
